# The dawn of surgical treatment of aortic insufficiency

**DOI:** 10.1111/jocs.16851

**Published:** 2022-08-18

**Authors:** Igor Vendramin, Uberto Bortolotti, Aldo D. Milano, Ugolino Livi

**Affiliations:** ^1^ Cardiothoracic Department University Hospital Udine Italy; ^2^ Division of Cardiac Surgery University Hospital Bari Italy

**Keywords:** aorta and great vessels

“Failure is success in progress”

‐ *Albert Einstein (1879−1955)*

Before the development and introduction in the clinical practice of the heart‐lung machine in 1953, to allow intracardiac procedures to be performed under cardiopulmonary bypass (CPB),^1^ certain cardiac operations could be accomplished only on a beating heart under mild hypothermia or with the use of cross‐circulation, as utilized by Walton C. Lillehei to successfully repair even complex congenital heart malformations.^2^

In 1953, Hufnagel (Figure 1) and Harvey reported the successful implantation of a ball valve prosthesis into the thoracic aorta^3^ (Figure 2). This historical operation was performed on September 11, 1952 at Georgetown University Hospital in Washington, DC, in a female patient with severe aortic valve insufficiency.^4^ This device, designed to replicate the mechanism of a liquor bottle stopper, produced almost one century ago,^3^ consisted in a tubular chamber, with an inlet and an outlet, containing a hollow ball to reduce its gravity; indeed, a pressure of just 5 mmHg was enough to move the poppet in a completely open or closed position.^5^ The whole device was molded from a single piece to obtain a smooth surface. Initially, the entire prosthesis was made of methyl methacrylate (Lucite); subsequently the ball was changed with one made by a hollow nylon core covered by silicone rubber to reduce prosthetic noise.

**Figure 1:**
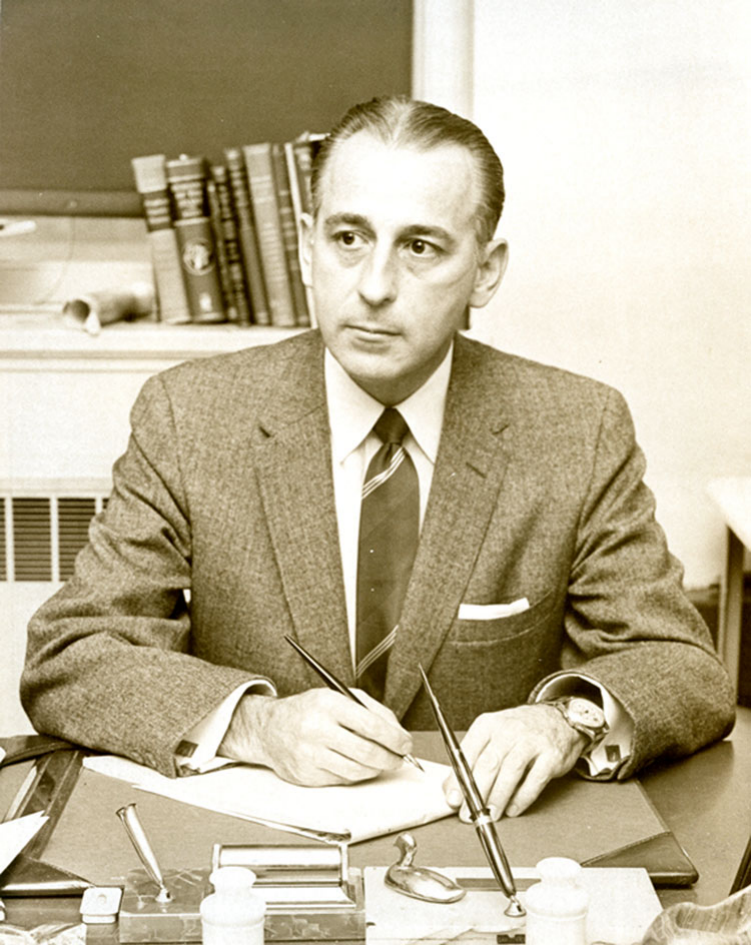
Charles A. Hufnagel (1916−1989) (reproduced with permission from the Georgetown University Archives)

**Figure 2:**
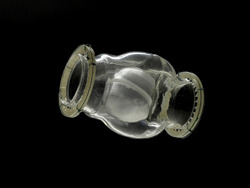
Original Hufnagel's ball valve prosthesis (reproduced with permission by Division of Medicine and Science, National Museum of American History, Smithsonian Institution, Washington, DC).

As Hufnagel himself stated: “*This valve was developed for the treatment of aortic insufficiency and to serve as a prototype to test the possibility that a valvular prosthesis would satisfactorily function within the cardiovascular system*.”^6^ In those years the CPB machine was still unavailable while replacement of the ascending aorta had not yet been performed. Therefore, Hufnagel was forced to insert this device into the descending aorta and implanting a prosthesis in that location was certainly made possible by the demonstration that the thoracic aorta could be safely temporarily clamped, as occurred during the first landmark operations performed by Robert Gross to close a patent ductus arteriosus or repair an aortic coarctation.^7^, ^8^

The operation to implant the Hufnagel prosthesis was performed through a standard posterolateral thoracotomy incision through the 5th intercostal space with the patient placed in the right lateral decubitus. As described by Hufnagel himself, the prosthesis was implanted in the descending aorta just below the takeoff of the left subclavian artery.^5^ Toinsert the prosthesis (Figure 3), following proximal and distal cross‐clamping, a transverse segment of the descending thoracic aorta was excised and the prosthesis inserted into both cut ends of the aorta; the prosthesis was fixed in place using flexible rings at the grooves present on the outer surface at both ends of the valve; occasionally, at the end of the procedure the aorta was wrapped with fabric material. Details of the operation, with some technical modifications, have also been described in the report by Conklin et al.^9^ Duration of valve implantation was generally performed in less than 10 min since aortic clamping >15 min was considered unsafe.^6^

**Figure 3:**
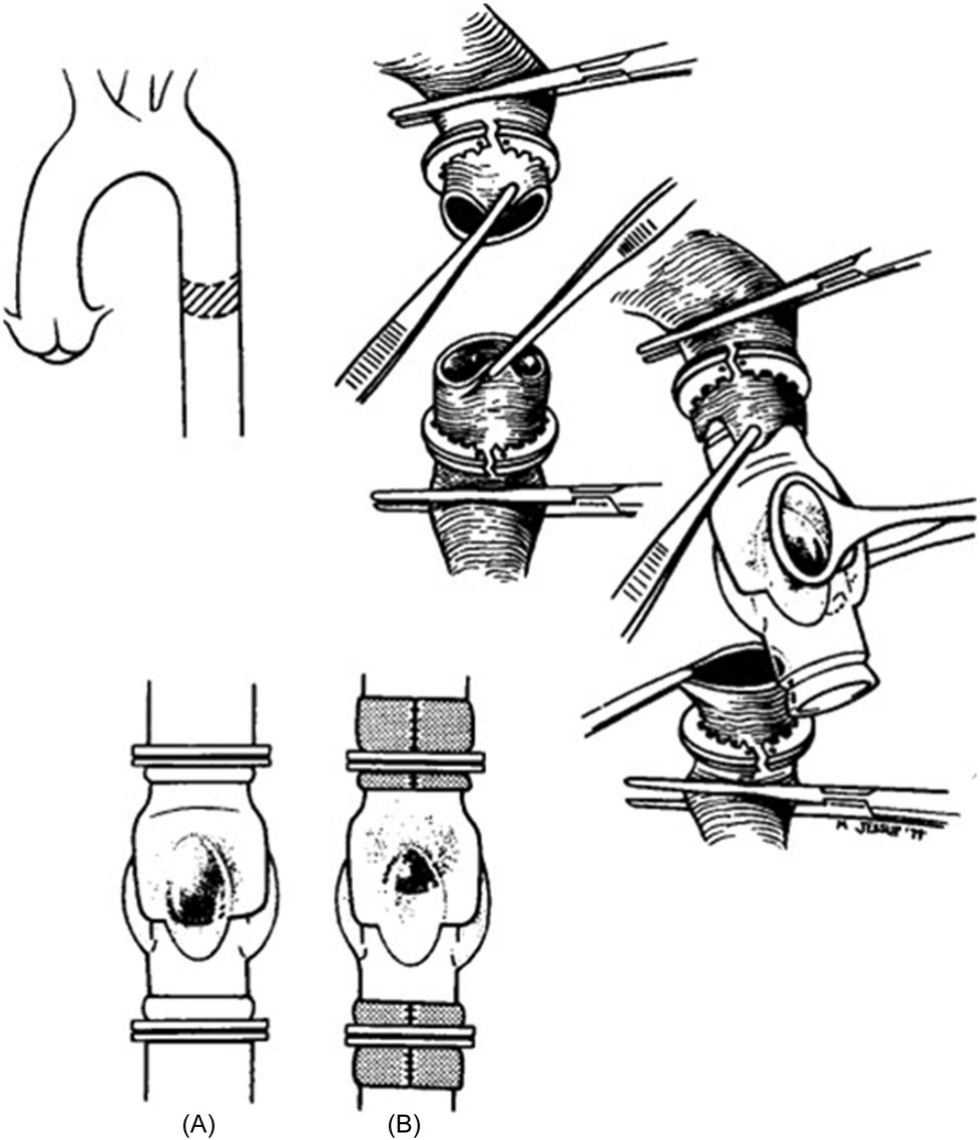
Drawing of the implantation technique of the Hufnagel prosthesis (reproduced with permission from Reference #10). (A) prosthesis in place fixed with flexible rings at both extremities; (B) Both prosthetic ends were at times reinforced with fabric.

Hufnagel reported in 1976 a series of 5 patients operated between 1953 and 1959.^6^ Four of these patients underwent subsequently prosthetic aortic valve replacement in the subcoronary position, but the original Hufnagel prosthesis was reported to function well up to 21 years without significant complications related to the original device.

In 1975, Fishbein and Roberts described the clinical and pathological observations in 2 patients who died between 11 and 13 years following implantation of a Hufnagel prosthesis.^10^ Despite the extended survival they did not find any signs of thrombosis, ball variance or hemolysis at necropsy. Based on these findings, they suggested that replacing the Hufnagel prosthesis at future operations on the aortic valve would have been unwise.^10^

The Hufnagel caged‐ball prosthesis was viewed more like an aortic‐assist device, since it did not replace the aortic valve, but was mainly designed with the aim of assisting patients with significant aortic valve incompetence; indeed, it reduced significantly aortic regurgitation but could not completely control “*all of the aortic leaks*” because the poorly functioning native aortic valve remained untouched; nevertheless, in many patients clinical improvement occurred even at long‐term follow‐up.^6^

Interestingly, 40 years later, the Hufnagel principle was revitalized by Cale et al.^11^ who reported 4 patients with malfunction of biological prostheses following aortic valve replacement who were considered at extremely high risk for reoperation or even inoperable; in all of them a mechanical prosthesis was implanted into the thoracic aorta with clinical improvement up to 6 years postoperatively.

The first aortic and mitral caged‐ball mechanical prostheses were implanted in orthotopic position in 1960 and dramatically changed the outlook of patients with cardiac valve disease.^12^, ^13^ This year marks the 70th anniversary of the first implantation of an artificial prosthesis as performed by Hufnagel to relieve aortic valve incompetence. This milestone operation, and those which followed shortly thereafter, demonstrate the courage of talented pioneers which, after a long and exciting journey full of hopes and realities, failures, and successes, allowed cardiac surgery to reach today results certainly unexpected seven decades ago. Therefore, the new generations must be grateful to all the work done in the past few years if at present, starting from the first caged‐ball prosthesis model, they can have available extremely reliable cardiac valve substitutes for an optimal care of their patients.

## CONFLICT OF INTEREST

The authors declare no conflict of interest.
